# Supplementary material for: Midbrain ghrelin receptor signalling regulates binge drinking in a sex specific manner
Source: Nat Commun. 2025 Mar 15;16:2568. doi: 10.1038/s41467-025-57880-w (PMC11910522; doi:10.1038/s41467-025-57880-w)
Supplement: Supplementary file 1 — Supplementary Information [file 41467_2025_57880_MOESM1_ESM.pdf]

SUPPLEMENTAL FIGURES – Pearl & Maddern et al.

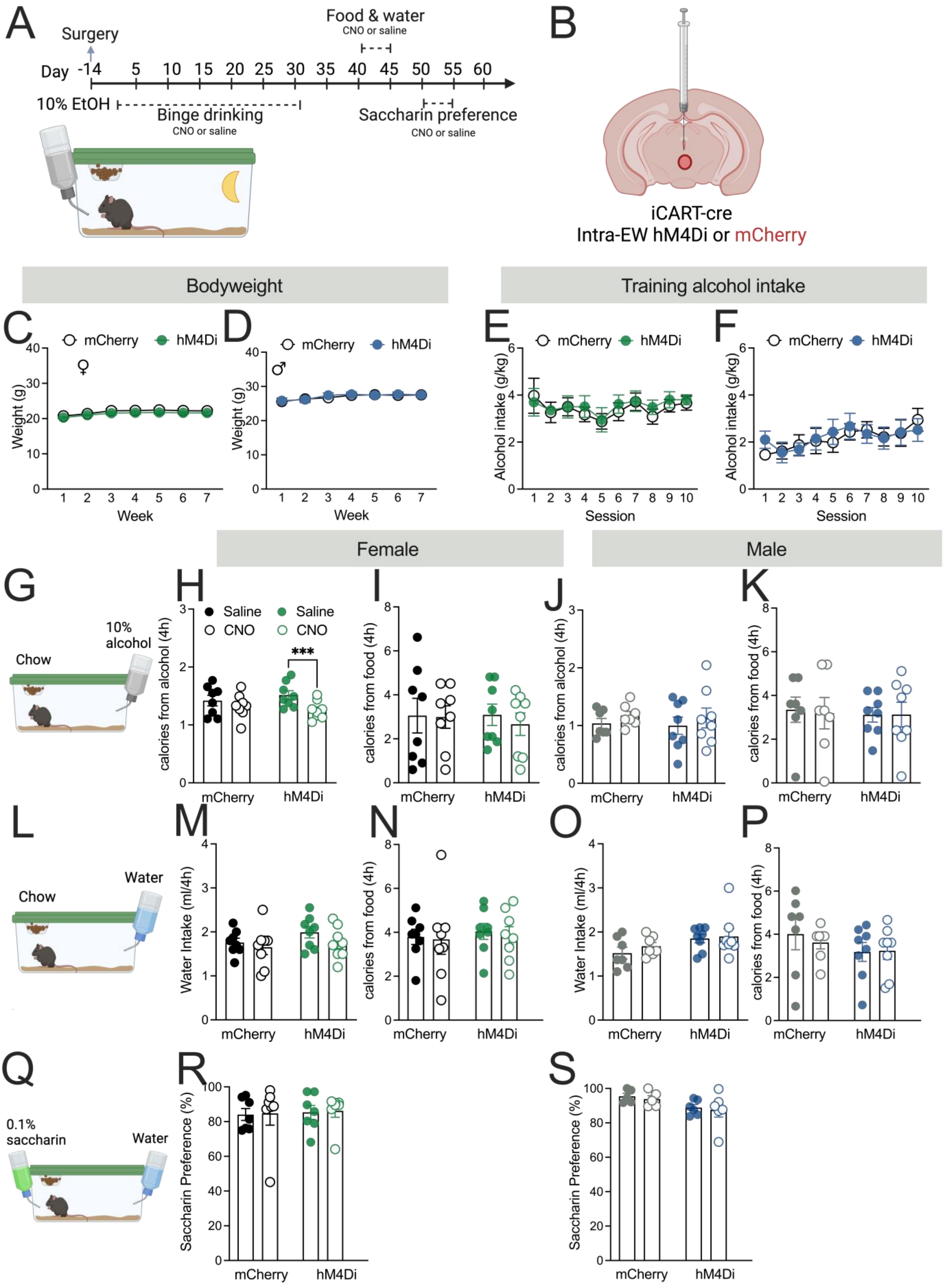

Supplementary Figure 1, related to Figure 1: Chemogenetic inhibition of EW^{CART} cells does not alter weight, water, food or saccharin intake. (A) Schematic of experimental outline and **(B)** viral strategy. RM two-way ANOVA showed no difference in weight of **(C)** female (virus F (1, 15) = 0.8869, *p*=0.3612; or interaction F (6, 90) = 0.2192, *p*=0.9697; but a main effect of time F (6, 90) = 24.09, *p*<0.0001; n=8/group), or **(D)** male mice (virus F (1, 13) = 0.04279, *p*=0.8393; or Interaction F (6, 78) = 1.094, *p*=0.3736; but a main effect of time (F (6, 78) = 26.51, *p*<0.0001; n=7 mCherry, 8 hM4Di) between hM4Di and control virus treated mice. Further during training no difference in alcohol consumption was observed in **(E)** female (RM two-way ANOVA, virus F (1, 15) = 0.1544, *p*=0.6999; interaction F (9, 135) = 0.1727, *p*=0.9965; or time F (9, 135) = 1.303, *p*=0.2407; n=8/group) or **(F)** male mice (RM two-way ANOVA, virus F (1, 13) = 0.01091, *p*=0.9184; or F (9, 117) = 1.211, *p*=0.2947; but a main effect of time F (9, 117) = 6.673, *p*<0.0001; n=7 mCherry, 8 hM4Di). **(G)** Schematic of binge drinking. **(H)** Female mice showed a specific reduction in Kcal intake from alcohol (RM two-way ANOVA, no effect virus F (1, 14) = 0.01532, *p*=0.9033; main effect CNO F (1, 14) = 26.96, *p*=0.0001; and interaction F (1, 14) = 6.018, *p*=0.0279; n=8/group). Bonferroni post-hoc between saline and CNO treated mice showed this was specific to hM4Di mice (*p*=0.0002), not mCherry (*p*=0.1464). **(I)** Further no differences in food intake with alcohol (RM two-way ANOVA, no effect virus F (1, 14) = 0.03906, *p*=0.8462; CNO F (1, 14) = 0.4057, *p*=0.5345; or interaction F (1, 14) = 0.2224, *p*=0.6445; n=8/group) were observed in female mice. **(J)** No difference in Kcal intake from alcohol in male mice (RM two-way ANOVA, no effect virus, F (1, 13) = 0.06261, *p*=0.8063; CNO F (1, 13) = 2.530, *p*=0.1357; or interaction F (1, 13) = 0.0005652, *p*=0.9814; n=7 mCherry, 8 hM4Di), or **(K)** food intake with alcohol (RM two-way ANOVA, virus F (1, 13) = 0.04826, *p*=0.8295; CNO F (1, 13) = 0.03520, *p*=0.8541; or interaction F (1, 13) = 0.04677, *p*=0.8321; n=7 mCherry, 8 hM4Di). **(L)** Schematic of food and water intake. No difference in **(M)** water intake (RM two-way ANOVA, virus F (1, 14) = 1.213, *p*=0.2892; CNO F (1, 14) = 2.395, *p*=0.1440; or interaction F (1, 14) = 0.3441, *p*=0.5668; n=8/group) or **(N)** Kcal food intake (RM two-way ANOVA,; virus F (1, 14) = 0.1538, *p*=0.7008; CNO F (1, 14) = 0.1489, *p*=0.7054; or interaction F (1, 14) = 0.01001, *p*=0.9217; n=8/group) was observed in female mice. **(O)** A trend towards main effect of virus was observed in male mice on water intake (RM two-way ANOVA, virus F (1, 13) = 3.440, *p*=0.0865; no effect CNO F (1, 13) = 0.9864, *p*=0.3387; or interaction F (1, 13) = 0.2832, *p*=0.6036), but no difference between CNO and saline with Bonferroni *post hoc,* mCherry, *p*=0.6307; hM4Di, *p*>0.9999; n=7 mCherry, 8 hM4Di), and no effect on **(P)** Kcal food intake with water (RM two-way ANOVA, virus F (1, 13) = 1.017, *p*=0.3317; CNO F (1, 13) = 0.2711, *p*=0.6114; or interaction F (1, 13) = 0.4660, *p*=0.5068; n=7 mCherry, 8 hM4Di) was observed in male mice. **(Q)** Schematic of saccharin preference. No difference in 0.1% saccharin preference was observed in **(R)** female (RM two-way ANOVA, virus F (1, 12) = 0.06909, p=0.7971; CNO F (1, 12) = 0.03693, *p*=0.8508; or interaction F (1, 12) = 0.001049, *p*=0.9747; n=7/group) mice. **(S)** Male mice with hM4Di DREADD showed greater intake than mCherry mice (main effect virus F (1, 9) = 5.219, *p*=0.0482), however, there was no effect of CNO (F (1, 9) = 0.3088, *p*=0.5920; or interaction F (1, 9) = 0.003404, *p*=0.9548; n=5 mCherry, 6 hM4Di). Bonferroni *post hoc* showed no difference between saline and CNO for mCherry (*p*>0.9999) or hM4Di *p*>0.9999). Data expressed as mean ± SEM. Source data are provided as a source data file. Created in BioRender. Walker, L. (2025) https://BioRender.com/l12b283 & https://BioRender.com/a19p668 [Agreement #RQ27UEVW56 & NH27UEW4CZ].

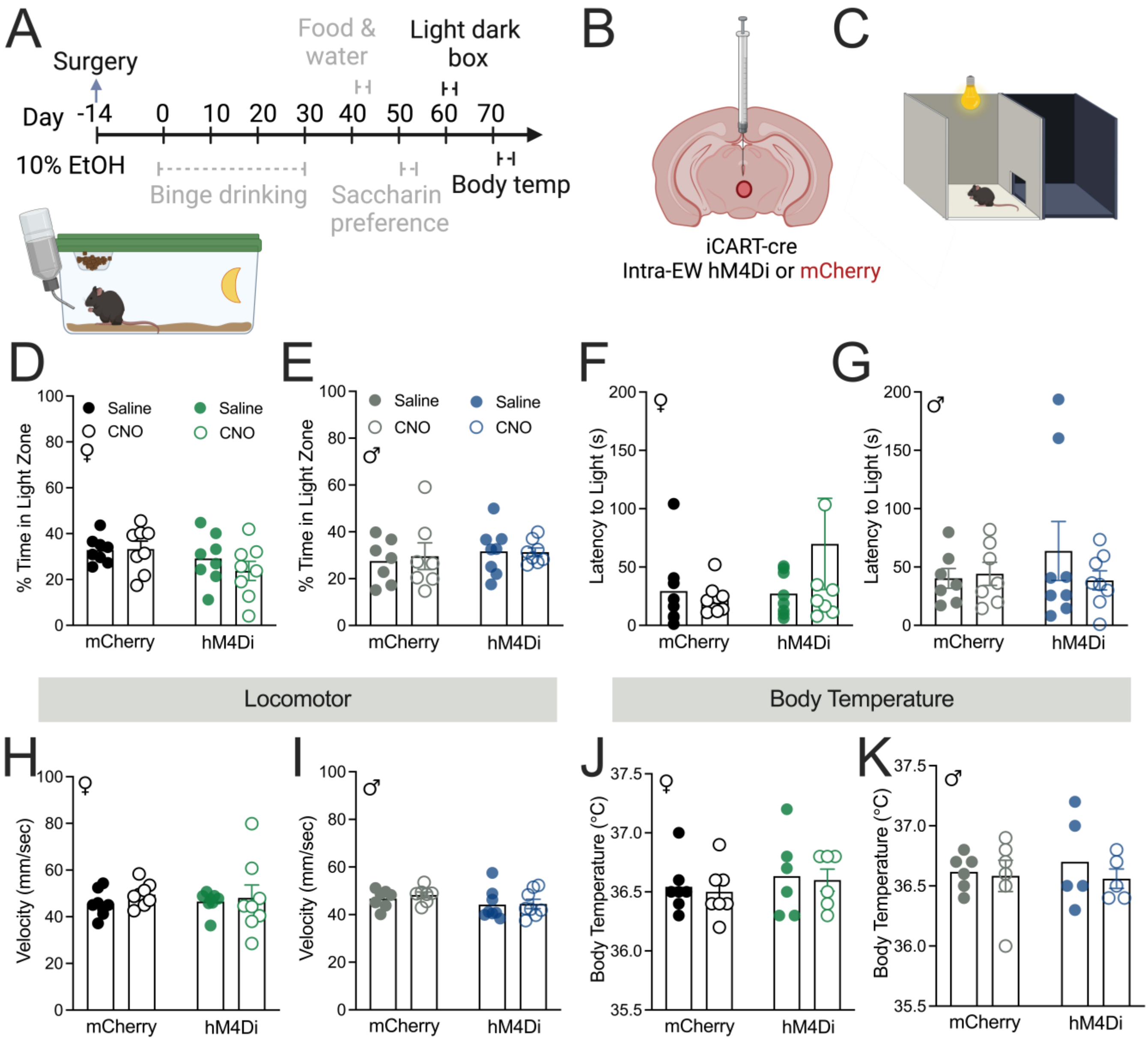

Supplementary Figure 2, related to Figure 1: Chemogenetic inhibition of EW^{CART} cells does not alter anxiety-like behaviour or body temperature. (A) Experimental timeline, **(B)** viral strategy and **(C)** schematic of light-dark box. Two-way repeated measures ANOVA showed chemogenetic inhibition of EW^{CART} cells did not alter % time spent in the light zone of **(D)** female (virus F (1, 15) = 2.394, *p*=0.1426; CNO F (1, 15) = 0.4651, *p*=0.5056; or interaction F (1, 15) = 0.6733, *p*=0.4248; n=8/group) or **(E)** male control or DREADD treated mice (virus F (1, 13) = 0.3790, *p*=0.5488; CNO F (1, 13) = 0.1344, *p*=0.7198; or interaction F (1, 13) = 0.2504, *p*=0.6252; n=7mCherry, 8 hM4Di); latency to light in **(F)** female (virus F (1, 15) = 0.9889, *p*=0.3358; CNO F (1, 15) = 0.4883, *p*=0.4954; or interaction F (1, 15) = 1.025, *p*=0.3273; n=8/group) or **(G)** male control or DREADD treated mice (virus F (1, 13) = 0.3208, *p*=0.5807; CNO F (1, 13) = 0.4987, *p*=0.4926; or interaction F (1, 13) = 0.9266, *p*=0.3533; n=7mCherry, 8 hM4Di). Further RM two-way ANOVA showed no effect of CNO on velocity in **(H)** female (virus F (1, 15) = 0.08511, *p*=0.7745; CNO F (1, 15) = 0.4294, *p*=0.5222; or interaction F (1, 15) = 0.3438, *p*=0.5664; n=8/group) or **(I)** but a trend towards main effect of virus was observed in male mice (virus F (1, 13) = 3.320, *p*=0.0915; CNO F (1, 13) = 0.2324, *p*=0.6378; or interaction F (1, 13) = 0.09966, *p*=0.7572. Bonferroni *post hoc* showed no difference between saline and CNO treatment for mCherry (*p*=0.8353) or hM4Di (*p*=0.9910) mice (n=7mCherry, 8 hM4Di). No effect on body temperature in **(J)** female (RM two-way ANOVA, virus F (1, 11) = 0.5349, *p*=0.4798; CNO F (1, 11) = 0.4206, *p*=0.5299; or interaction F (1, 11) = 0.006572, *p*=0.9368; n=7 mCherry, 6 hM4Di) or **(K)** male control or DREADD treated mice (RM two-way ANOVA, virus F (1, 9) = 0.03924, *p*=0.8474; CNO F (1, 9) = 1.819, *p*=0.2103; or interaction F (1, 9) = 0.6890, *p*=0.4280; n=6 mCherry, 5 hM4Di). Data expressed as mean ± SEM, n = 6-9/group. Source data are provided as a source data file. Created in BioRender. Walker, L. (2025) https://BioRender.com/p75g008 & https://BioRender.com/v56y748 [Agreement #ZX27UEWEJG & VO27UEWR8I].

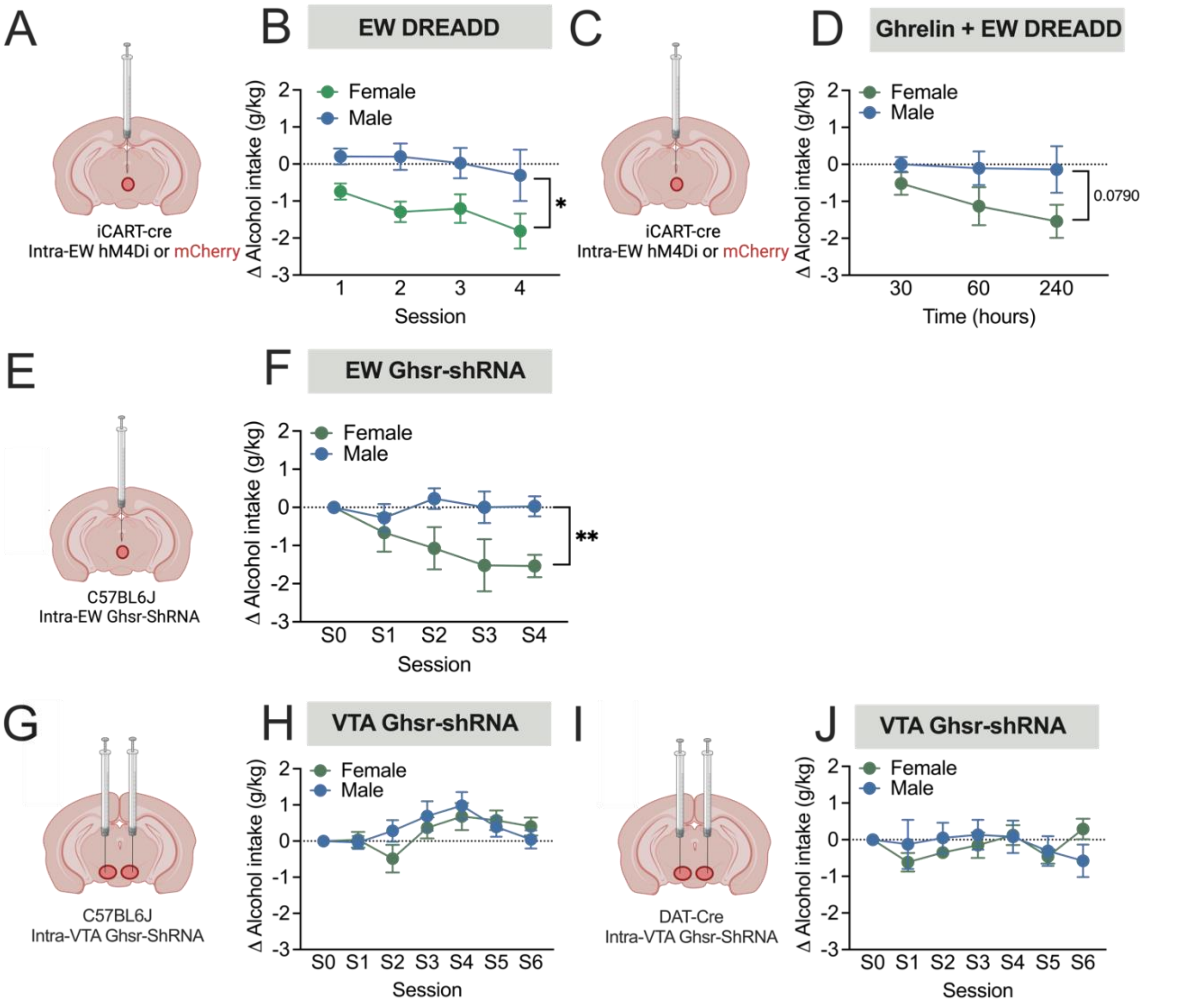

Supplementary Figure 3, related to Figure 1, 2 and Supp 3: EW inhibition specifically reduces alcohol consumption in female mice. (A) Schematic of viral strategy. **(B)** Female C57BL6J mice with hM4Di in the EW showed a specific reduction in delta (Δ) alcohol intake when treated with CNO compared to male mice (RM two way ANOVA, sex F (1, 15) = 7.874, p=0.0133; time F (3, 45) = 2.977, p=0.0414; no interaction F (3, 45) = 0.4816, p=0.6967; Bonferroni post hoc female vs. male S1 p=0.3966, S2 p=0.0429, S3 p =0.1357, S4 p=0.0399, n=9F, 8M). **(C)** Schematic of viral strategy. **(D)** Female C57BL6J mice with hM4Di in the EW showed a specific reduction in Δ ghrelin-induced alcohol intake when treated with CNO compared to male mice (RM two-way ANOVA, trend towards main effect, sex F (1, 14) = 3.590, p=0.0790; no effect of session F (1.477, 20.68) = 1.766, p=0.1997; or interaction F (2, 28) = 1.003, p=0.3797; n = 8/sex). **(E)** Schematic of viral strategy. **(F)** Female mice with Ghsr-ShRNA injected in the EW showed a specific reduction in Δ alcohol intake compared to male mice (RM two-way ANOVA, main effect of sex F (1, 12) = 9.582, p=0.0093; no effect of session F (2.786, 33.43) = 1.446, p=0.2479; or interaction F (4, 48) = 1.908, p=0.1244; Bonferroni *post hoc female* vs. male S1 p=0.9788, S2 p=0.2859, S3 p=0.3607, S4 p=0.0092; n =7/sex). **(G)** Schematic of viral strategy. **(H)** No difference in Δ alcohol intake was observed between sexes when Ghsr-ShRNA was injected in the VTA of C57BL6J mice (RM two-way ANOVA, no effect sex F (1, 16) = 0.1393, p=0.7139; interaction F (6, 96) = 1.396, p=0.2242; main effect of session F (6, 96) = 4.569, p=0.0004; n=11F, 7M). **(I)** Schematic of viral strategy. **(J)** No difference in Δ alcohol intake was observed between sexes when Ghsr-ShRNA was injected in the VTA of DAT-Cre mice (RM two-way ANOVA, no main effect sex, F (1, 9) = 0.04053, p=0.8449; session F (3.188, 28.69) = 0.8519, p=0.4829, or F (6, 54) = 1.260, p=0.2914; n=6F, 7M). Data expressed as mean ± SEM. Source data are provided as a source data file. Created in BioRender. Walker, L. (2025) https://BioRender.com/e95n462 [Agreement #RW27UEX5ES].

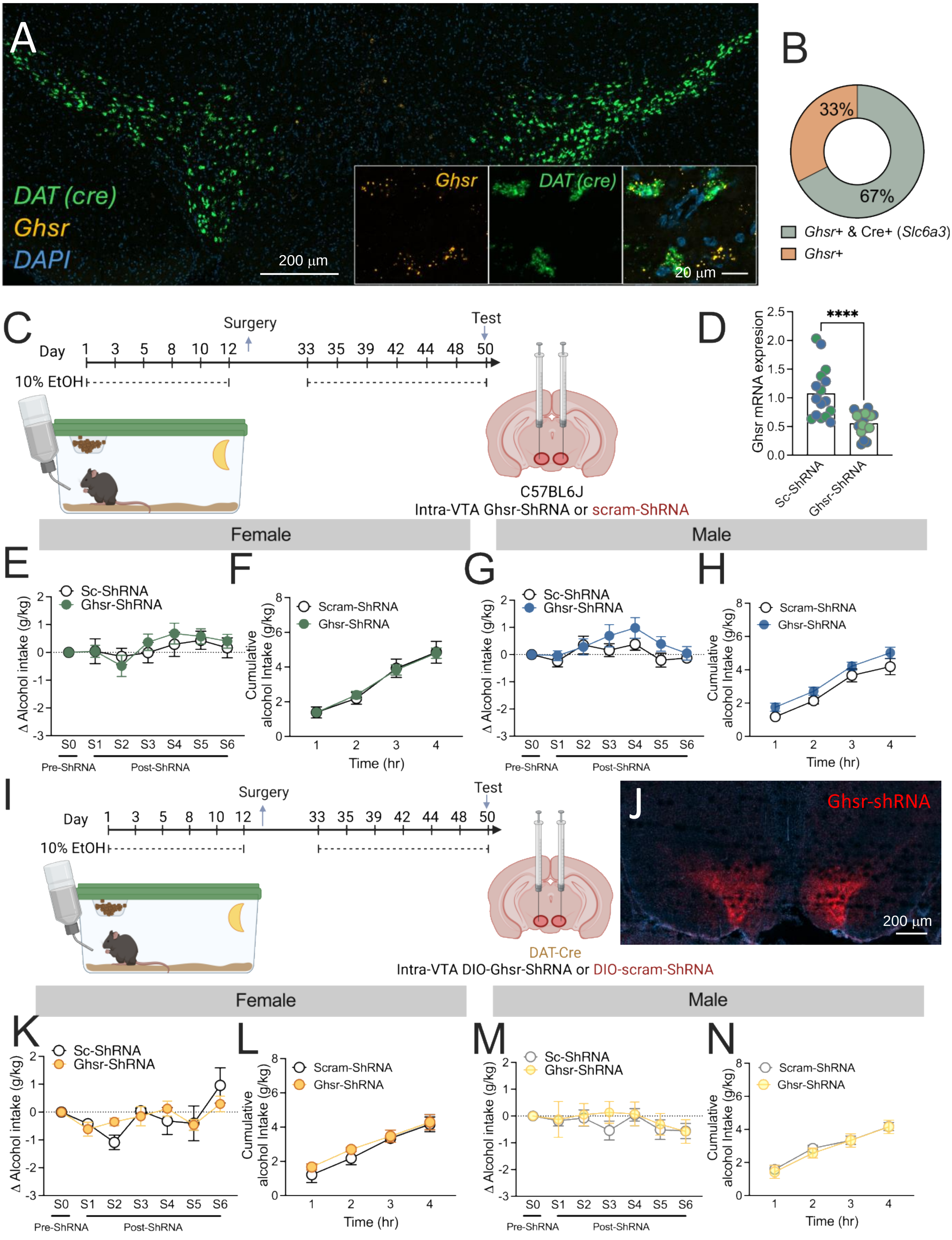

Supplementary Figure 4, VTA GHSR knockdown does not alter binge drinking in male or female mice. (A) Representative image of Ghsr expression with DAT (cre) in the VTA. **(B)** Ghsr mRNA was expressed in 67% Cre+ (DAT) cells in the VTA, but also on 33% DAT-negative cells. **(C)** Schematic of viral

strategy. **(D)** Ghsr-ShRNA reduced *Ghsr* mRNA expression in the VTA (unpaired t-test, t=4.450, df=32, p<0.0001; n=15 sc-ShRNA [7F, 8M pooled], 18 *Ghsr*-ShRNA [11F, 7M pooled]). **(E)** No significant difference was observed in female mice post shRNA knockdown during training (Two-way ANOVA, no effect of treatment F (1, 17) = 0.1594, p=0.6947; interaction F (11, 187) = 0.4879, p=0.9093; main effect of session F (4.962, 84.35) = 2.883, p=0.0191; Bonferroni *post hoc* showed no difference to S0 in Sc-ShRNA or Ghsr-ShRNA, p's>0.1) or **(F)** cumulative intake during test (Two-way ANOVA, no effect of treatment F (1, 17) = 0.0008908, p=0.9765; interaction F (3, 51) = 0.2372, p=0.8700; main effect of time F (3, 51) = 140.3, p<0.0001). **(G)** No significant difference was observed in male mice post shRNA knockdown during training (Two-way ANOVA, no effect of treatment F (1, 14) = 2.262, p=0.1548; interaction F (11, 154) = 1.335, p=0.2102; main effect of session F (4.523, 63.32) = 7.010, p<0.0001; Bonferroni *post hoc* compared to S0, Sc-ShRNA S0 vs. S3 p=0.0039, S0 vs. S10 p=0.0640; Ghsr-ShRNA S0 vs S6 p=0.0092, S0 vs. S10 p=0.0669) but a trend in **(H)** cumulative intake during test (Two-way ANOVA, treatment F (1, 13) = 3.150, p=0.0993; main effect of time F (3, 39) = 98.46, p<0.0001; no interaction F (3, 39) = 0.2024, p=0.8941). **(I)** Schematic of viral strategy. **(J)** Representative image of Ghsr-ShRNA in the VTA of DAT-Cre mice. **(K)** No significant difference was observed in female DAT-Cre mice post shRNA knockdown during training (Two-way ANOVA, treatment F (1, 9) = 0.001528, p=0.9697; or interaction F (6, 54) = 1.275, p=0.2845; main effect of session F (2.405, 21.65) = 4.730, p=0.0153; Bonferroni *post hoc* showed no difference to S0 in Sc-ShRNA or Ghsr-ShRNA, *p's*>0.1) or **(L)** cumulative intake during test (Two-way ANOVA, treatment F (1, 17) = 0.0008908, p=0.9765; interaction F (3, 51) = 0.2372, p=0.8700; main effect time F (3, 51) = 140.3, p<0.0001). **(M)** No significant difference was observed in male DAT-Cre mice post shRNA knockdown during training (Two-way ANOVA, no effect treatment F (1, 10) = 0.1883, p=0.6735; session F (3.158, 31.58) = 1.615, p=0.2038; or interaction F (6, 60) = 0.4416, p=0.8481) or **(N)** cumulative intake during test (Two-way ANOVA, no effect of treatment F (1, 10) = 0.1027, p=0.7552; interaction F (3, 30) = 0.2577, p=0.8552; main effect time F (3, 30) = 73.20, p<0.0001). Data expressed as mean ± SEM. n = 7 female C57BL6J Sc-ShRNA, 12 female C57BL6J Ghsr-ShRNA, 9 male C57BL6J Sc-ShRNA, 7 female C57BL6J Ghsr-ShRNA; n = 5 female DAT-Cre Sc-ShRNA, 6 female DAT-Cre Ghsr-ShRNA, 7 male DAT-Cre Sc-ShRNA, 5 male DAT-Cre Ghsr-ShRNA. Source data are provided as a source data file. Created in BioRender. Walker, L. (2025) https://BioRender.com/u91y968 & https://BioRender.com/q38o338 [Agreement # FF27UEXDWN & GX27UEXK25].

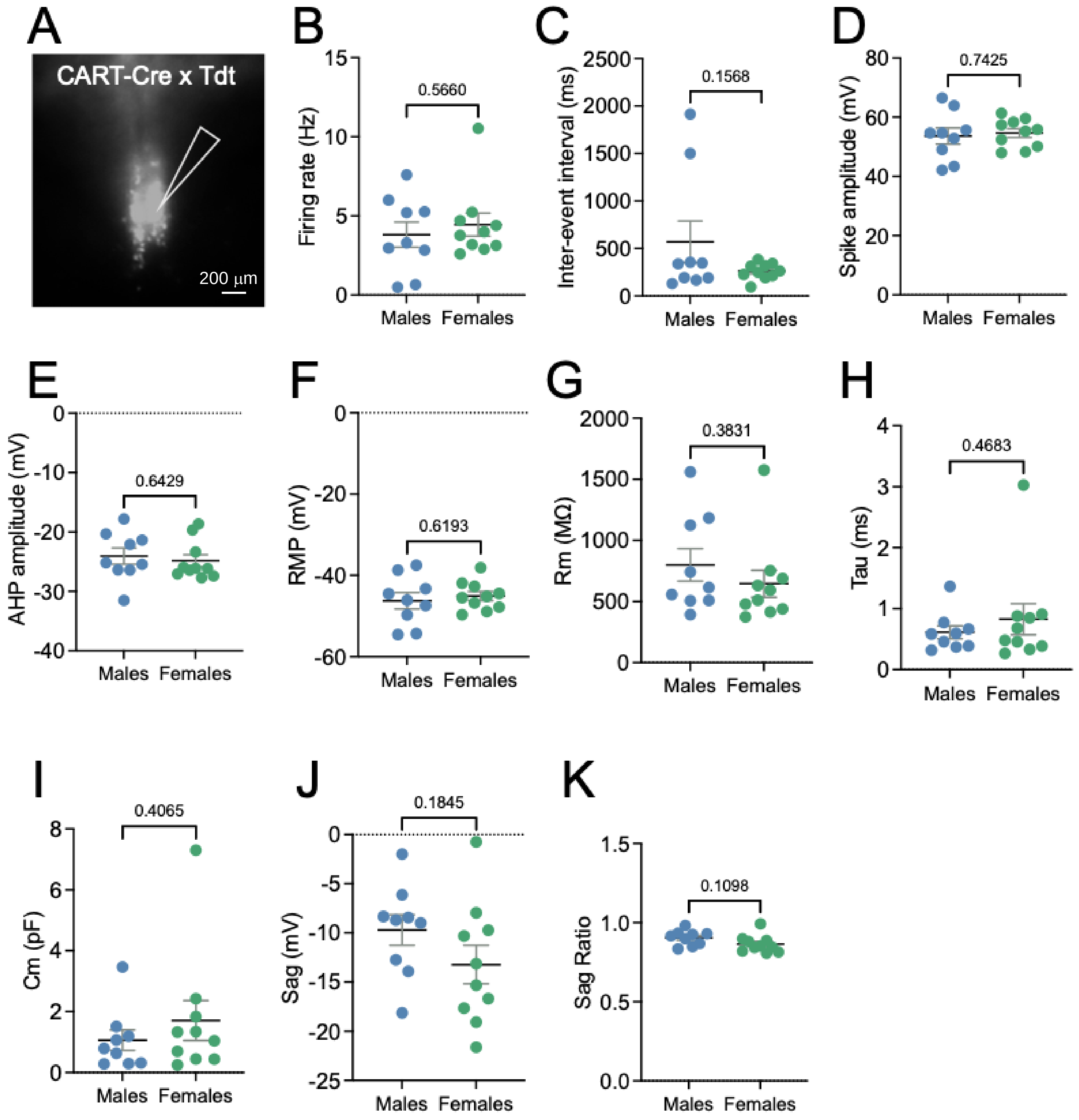

Supplementary Figure 5, related to Figure 4: No sex difference in basal electrophysiological properties of EW^{CART} cells. (A) overview of Tdtomato expression in the EW where patch clamp recording were collected. Unpaired t-test showed no sex differences were in **(B)** Firing rate (t=0.5854, df=17, *p*=0.5660), **(C)** inter-event interval (t=1.482, df=17, *p*=0.1568), **(D)** spike amplitude (t=0.3339, df=17, *p*=0.7425), **(E)** AHP amplitude (t=0.4720, df=17, *p*=0.6429), **(F)** resting membrane potential (RMP, t=0.5060, df=17, *p*=0.6193), **(G)** membrane resistance (Rm, t=0.8953, df=17, *p*=0.3831), **(H)** tau (t=0.7418, df=17, *p*=0.4683), **(I)** membrane time constant (Cm, t=0.8511, df=17, *p*=0.4065), **(J)** sag (t=1.383, df=17, *p*=0.1845), or **(K)** sag ratio (t=1.687, df=17, *p*=0.1098) when directly comparing male and female mice. Data expressed as mean ± SEM. n = 10 cells from 7 female mice, 9 cells from 7 male mice. Source data are provided as a source data file.

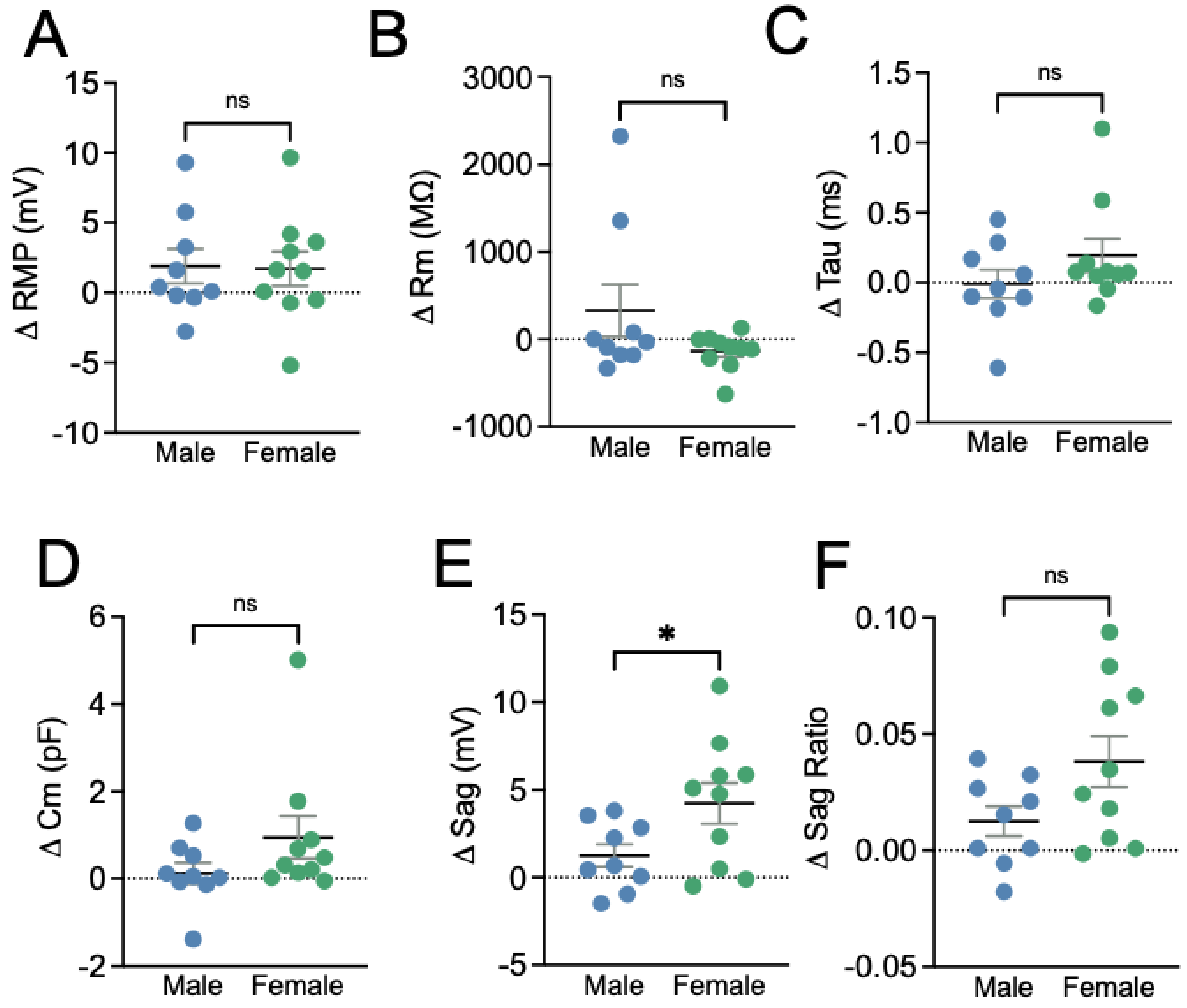

Supplementary Figure 6, related to Figure 4: Sex differences in firing properties of EW^{CART} cells following ghrelin application. Unpaired t-test showed no sex differences in ghrelin induced changes in **(A)** resting membrane potential (RMP, t=0.1051, df=17, *p*=0.9175), **(B)** membrane resistance (Rm, t=1.589, df=17, *p*=0.1305), **(C)** tau (t=1.297, df=17, p=0.2118), **(D)** membrane time constant (Cm, t=1.479, df=17, *p*=0.1576), a significant difference in **(E)** sag (t=2.179, df=17, *p*=0.0437), and trend toward difference in **(F)** sag ratio (t=1.967, df=17, *p*=0.0658) following bath application of ghrelin. Data expressed as mean ± SEM. n = 10 cells from 7 female mice, 9 cells from 7 male mice. Source data are provided as a source data file.

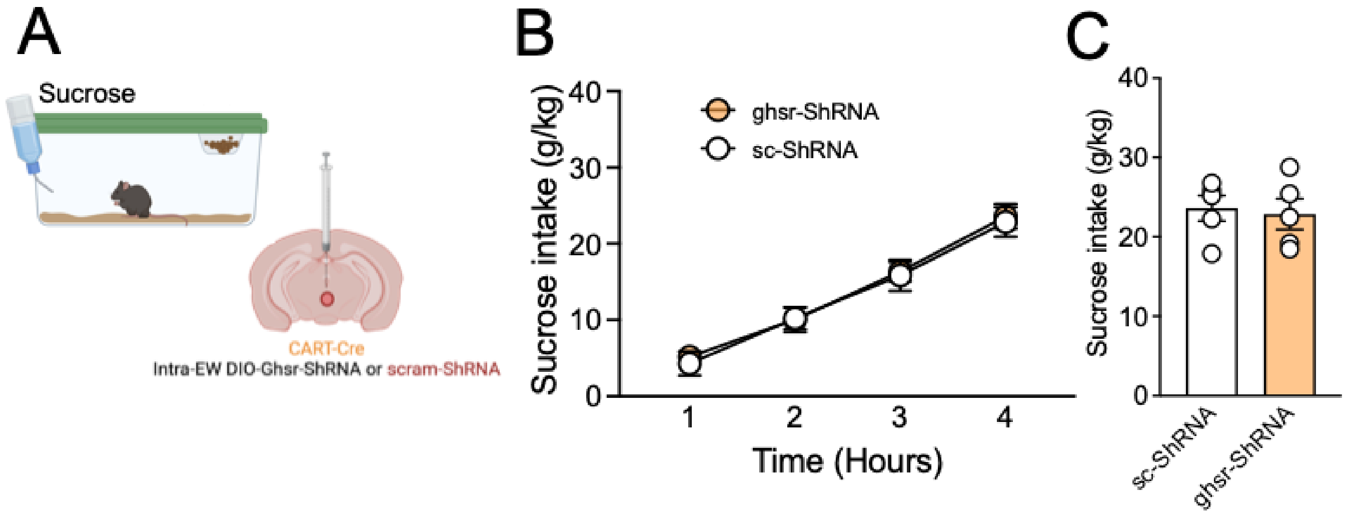

Supplementary Figure 7, related to Figure 5: GHSR knockdown from EW^{CART} cells does not alter sucrose intake. (A) Schematic of experiment outline of sucrose intake. No difference in **(B)** cumulative sucrose consumption over time (RM two-way ANOVA, virus F (1, 8) = 0.04907, p=0.8302; or interaction F (3, 24) = 0.5929, *p*=0.6257; but main effect of time F (3, 24) = 688.3, *p*<0.0001), or **(C)** total sucrose consumption (unpaired t-test, t=0.2934, df=8, *p*=0.7767) was observed in female mice with GHSR injected within the EW. Data expressed as mean ± SEM, n=5 mice/treatment. Source data are provided as a source data file. Created in BioRender. Walker, L. (2025) https://BioRender.com/n20k071 [Agreement # NM27UEXY8C].

SUPPLEMENTAL TABLES

Table S1: Key reagents

**Viruses**	**Supplier**		**Cat #**
AAV2-hsyn-DIO-hM4Di-mcherry	Addgene		44362-AAV2
AAV2-hsyn-DIO-mcherry	Addgene		50459-AAV2
AAV2-hsyn-Ghsr-ShRNA-mCherry	Vector Biolabs		This paper
AAV2-hsyn-scramble-ShRNA-mCherry	Vector Biolabs		This paper
AAV2-hSyn-DIO-Ghsr-ShRNA-mCherry	Vector Biolabs		This paper
AAV2-hSyn-DIO-scramble-ShRNA-mCherry	Vector Biolabs		This paper
**Drugs**	**Supplier**		**Cat #**
LEAP2 (37-76)	Phoenix pharmaceuticals		#075-58
JMV2959	MedChemExpress,		#HY-U00433A
Ghrelin (i.p)	BOC Sciences		B0084-103854
Ghrelin (bath)	BOC Sciences		B0084-103854
**Antibodies**	**Supplier**	**RRID**	**Cat #**
Rabbit anti-CART	Phoenix Pharmaceuticals	AB_2313614	H-003-62
Goat-anti-CART	R&D systems	AB_2068569	#AF163
Goat anti-cFos	Santa Cruz	AB_2629503	SC-52-G
Chicken anti-RFP	Rockland	AB_10704808	600-901-379
Donkey anti-rabbit AlexaFluor488	Life Technologies	AB_2535792	A-21206
Donkey anti-Goat- AlexaFluor594	Life Technologies	AB_2534105	A-11058
Donkey anti-Chicken 594	Jackson Immunology	AB_2340377	703-585-155
**RNAscope**	**Supplier**		**Cat #**
RNAscope Multiplex Fluorescent Kit (V1)	Advanced Cell Diagnostics		320850
Ghsr	Advanced Cell Diagnostics		426148
Cartpt	Advanced Cell Diagnostics		432008-C2
Slc17a6 (vGlut2)	Advanced Cell Diagnostics		319171-C3
Cre	Advanced Cell Diagnostics		#312281-C2

Table S2: Primer Sequences

	**Forward**	**Reverse**
Actin	GAACCCTAAGGCCAACCGTG	GGTACGACCAGAGGCATACA
Hprt	GCAGTACAGCCCCAAAATGG	GGTCCTTTTCACCAGCAAGCT
Ghsr	CTCAGGGACCAGAACCACAAAC	ACAAAGGACACCAGGTTGCAG
Esr1	CCTTGTCTCTTCCCTGATGTCAA	GTTCATTGTGACTGCCCTTGATC
Esr2	CGGTCTGTCTGAATGTGGTCA	GAAGCTGTGTGTGTGTGTGTC
Parq5	GTTACCGACACCCACAGAGTT	CGTCCAGATGTTGAGGGTCTC
Parq8	GACGACTGCCATCCTAGAGC	CTGCTGCCCACTCATTGACA

Table S3: Statistical analysis

Stats for Fig .1

	Analysis	Main effect: Sex		Treatment		Interaction	
b	2-tailed unpaired t-test			t=2.607, df=13	P=0.0217		
d	Two-way ANOVA	F (1, 28) = 6.567	P=0.0161	F (1, 28) = 26.86	P<0.0001	F (1, 28) = 1.477	P=0.2344
e	linear regression			R= 0.3877	P=0.0132		
f	Two-way ANOVA	F (1, 27) = 1.162	P=0.2907	F (1, 27) = 7.420	P=0.0112	F (1, 27) = 0.6193	P=0.4382
g	Two-way ANOVA	F (1, 26) = 0.7528	P=0.3935	F (1, 26) = 3.104	P=0.0899	F (1, 26) = 0.03023	P=0.8633
		**Time**		**Treatment**		**Interaction**	
k	RM Two-way ANOVA	F (3, 21) = 450.9	P<0.0001	F (1, 7) = 2.116	P=0.1891	F (3, 21) = 0.5561	P=0.6498
l	2-tailed Paired t-test			t=0.9523, df=7	P=0.3727		
m	RM Two-way ANOVA	F (3, 24) = 188.1	P<0.0001	F (1, 8) = 20.38	P=0.0020	F (3, 24) = 3.171	P=0.0426
n	2-tailed Paired t-test			t=3.378, df=8	P=0.0097		
o	RM Two-way ANOVA	F (3, 18) = 150.8	P<0.0001	F (1, 6) = 0.5880	P=0.4723	F (3, 18) = 0.9155	P=0.4532
p	2-tailed Paired t-test			t=0.1980, df=6	P=0.8496		
q	RM Two-way ANOVA	F (3, 21) = 95.49	P<0.0001	F (1, 7) = 0.003947	P=0.9517	F (3, 21) = 0.5557	P=0.6500
r	2-tailed Paired t-test			t=0.3474, df=7	P=0.7385		

Stats for Fig .2

		Analysis	Main effect: Time		Treatment		Interaction	
d		RM Two-way ANOVA	F (2, 42) = 94.15	P<0.0001	F (2, 21) = 5.364	P=0.0131	F (4, 42) = 1.457	P=0.2324
e		RM One-way ANOVA			F (2, 21) = 3.512	P=0.0483		
f		RM Two-way ANOVA	F (1.385, 9.693) = 51.95	P<0.0001	F (1.825, 12.77) = 0.02530	P=0.9674	F (2.41, 16.93) = 0.1348	P=0.9076
g		RM One-way ANOVA			F (2, 21) = 0.03941	P=0.9614		
j	EW	2-tailed unpaired t-test			t=5.729, df=23	P<0.0001		
	VTA	2-tailed unpaired t-test			t=0.8112, df=23	P=0.4256		
k		RM Two-way ANOVA	F (8, 88) = 2.301	P=0.0274	F (1, 11) = 1.853	P=0.2007	F (8, 88) = 2.500	P=0.0170
l		RM Two-way ANOVA	F (1.993, 21.92) = 189.7	P<0.0001	F (1, 11) = 5.328	P=0.0414	F (3, 33) = 1.558	P=0.2181
m		2-tailed unpaired t-test			t=2.527, df=11	P=0.0281		
n		linear regression			R2 = 0.4332	P=0.0145		
o		RM Two-way ANOVA	F (4.686, 46.86) = 2.884	P=0.0261	F (1, 10) = 2.639	P=0.1353	F (8, 80) = 1.006	P=0.4380
p		RM Two-way ANOVA	F (1.367, 13.67) = 56.69	P<0.0001	F (1, 10) = 0.2224	P=0.6473	F (3, 30) = 0.3392	P=0.7971
q		2-tailed unpaired t-test			t=0.2184, df=10	P=0.8315		
r		linear regression			R2 = 0.0001	P=0.9532		

Stats for Fig .3

	Analysis	Main effect: Time		Treatment		Interaction	
c	One way-ANOVA			F (2, 23) = 7.176	P=0.0038		
d	RM Two-way ANOVA	F (6, 102) = 7.570	P<0.0001	F (1, 17) = 0.8116	P=0.3802	F (6, 102) = 0.4490	P=0.8442
e	RM Two-way ANOVA	F (3, 51) = 171.1	P<0.0001	F (1, 17) = 0.003585	P=0.9530	F (3, 51) = 0.7051	P=0.5534
f	2-tailed unpaired t-test			t=0.6164, df=17	P=0.5458		
g	2-tailed unpaired t-test			t=3.871, df=17	P=0.0012		

Stats for Fig .4

		Analysis	stats	p value
d	Ghsr	2-tailed unpaired t-test	t=1.950, df=7	P=0.0922
	Cartpt	2-tailed unpaired t-test	t=0.7822, df=7	P=0.4598
	vGlut2	2-tailed unpaired t-test	t=1.207, df=7	P=0.2668
g		2-tailed unpaired t-test	t=2.232, df=9	P=0.0525
h		2-tailed unpaired t-test	t=3.850, df=9	P=0.0039
i		2-tailed unpaired t-test	t=2.628, df=9	P=0.0274
j		2-tailed unpaired t-test	t=1.296, df=9	P=0.2273
k		2-tailed unpaired t-test	t=0.1361, df=9	P=0.8947
m		2-tailed paired t-test	t=6.452, df=9	P<0.0001
m		2-tailed paired t-test	t=5.614, df=8	P=0.0005
n		2-tailed unpaired t-test	t=2.178, df=17	P=0.0438
n		2-tailed paired t-test	t=8.425, df=9	P<0.0001
o		2-tailed paired t-test	t=8.512, df=8	P<0.0001

p		2-tailed unpaired t-test	t=0.9445, df=17	P=0.3581

Stats for Fig .5		Main effect					
	Analysis	Time		Treatment		Interaction	
d	RM Two-way ANOVA	F (2.993, 47.88) = 4.752	P=0.0056	F (1, 16) = 9.113	P=0.0082	F (5, 80) = 0.9294	P=0.4665
e	RM Two-way ANOVA	F (1.664, 26.63) = 225.6	P<0.0001	F (1, 16) = 10.99	P=0.0044	F (3, 48) = 4.471	P=0.0076
f	2-tailed unpaired t-test			t=3.833, df=16	P=0.0015		
j	RM Two-way ANOVA	F (3.164, 53.78) = 1.317	P=0.2783	F (1, 17) = 0.002752	P=0.9588	F (5, 85) = 0.1803	P=0.9693
k	RM Two-way ANOVA	F (1.643, 27.92) = 188.0	P<0.0001	F (1, 17) = 0.8315	P=0.3746	F (3, 51) = 1.597	P=0.2015
l	2-tailed unpaired t-test			t=1.247, df=17	P=0.2293		

Stats for Fig .6			Main effect					
		Analysis	Time		Treatment		Interaction	
d		RM Mixed effects model	F (11, 109) = 1.481	P=0.1489	F (1, 10) = 0.1116	P=0.7453	F (11, 109) = 0.1950	P=0.9976
e	intra-EW	2-tailed Paired t-test			t=0.5968, df=6	P=0.5725		
	control	2-tailed Paired t-test			t=0.8929, df=4	P=0.4224		
f		RM Two-way ANOVA	F (1.471, 8.825) = 42.41	P<0.0001	F (1.431, 8.583) = 11.37	P=0.0058	F (2.760, 16.56) = 5.695	P=0.0082
g		RM One-way ANOVA			F (1.625, 9.749) = 11.60	P=0.0036		
h		RM Two-way ANOVA	F (1.270, 5.082) = 43.57	P=0.0009	F (1.748, 6.991) = 0.7170	P=0.5029	F (1.969, 7.876) = 1.674	P=0.2476
i		RM One-way ANOVA			F (1.127, 4.510) = 1.004	P=0.3797		
